# Associations of plant-based dietary patterns with cardiovascular risk factors in women

**DOI:** 10.34172/jcvtr.2022.01

**Published:** 2022-02-26

**Authors:** Zahra Shirzadi, Elnaz Daneshzad, Ahmadreza Dorosty, Pamela J Surkan, Leila Azadbakht

**Affiliations:** ^1^Department of Community Nutrition, School of Nutritional Sciences and Dietetics, Tehran University of Medical Sciences, Tehran, Iran; ^2^Non-Communicable Diseases Research Center, Alborz University of Medical Sciences, Karaj, Iran; ^3^Department of International Health, Johns Hopkins Bloomberg School of Public Health, Baltimore, USA; ^4^Diabetes Research Center, Endocrinology and Metabolism Clinical Sciences Institute, Tehran University of Medical Sciences, Tehran, Iran

**Keywords:** Vegetarians, Cardiovascular Diseases, Hyperlipidemias, Hypertriglyceridemic Waist, Obesity

## Abstract

***Introduction:*** Given that some plant-based foods, such as potatoes, adversely affect cardiovascular disease (CVD) risk factors, this study was performed to assess the association between plant dietary patterns and these risk factors.

***Methods:*** This cross-sectional study was conducted among 371 healthy 18 to 50 year-old Iranian women. Participant dietary intake was assessed using a validated food frequency questionnaire. Nineteen food groups were ranked in deciles and received scores from 1 to 10. An overall plant-based dietary index (PDI), a healthy plant-based dietary index (hPDI), and an unhealthy plant-based dietary index (uPDI) were calculated.

***Results:*** Participants who scored in the top tertile of the PDI or uPDI consumed less fat and protein and more carbohydrates, compared to women in the lowest tertile (*P* < 0.05). There was no significant variation in macronutrient consumption between the highest and lowest tertiles of hPDI. Participants who scored in the highest tertile of PDI had lower low density cholesterol level (LDL) (79.61 ± 14.36 mg dL−1 vs. 83.01 ± 14.96 mg/dL−1, *P* = 0.021). In addition, higher adherence to uPDI was associated with higher triglyceride (TG) levels compared to participants with lower adherence (101.5 ± 56.55 mg/dL−1 vs. 97.70 ± 56.46 mg dL−1, *P* < 0.0001). Here was no significant association between PDI, hPDI and uPDI and CVD risk factors in regression model.

***Conclusion:*** We found no significant association between plant-based dietary indices and CVD risk factors in women, except for LDL-C and TG. Future cohort studies are needed to confirm these findings.

## Introduction

Although substantial efforts have been made to reduce the prevalence of cardiovascular diseases (CVDs) worldwide, such disorders are the leading causes of death. Every year 17.9 million people die from CVD, which corresponds to 31% of all global mortality.^1^ Forty-six percent of Iranian deaths are due to CVDs. Also, the prevalence of some CVD risk factors such as hypertension, obesity, and hyperlipidemia are now high in Iran.^2^ Some plant foods are associated with cardiovascular outcomes; a systematic review and meta-analysis by Zurbau et al showed that fruits and vegetables have cardiovascular benefits.^3^ Another meta-analysis by Aune et al revealed that whole grains were associated with reduced risk of CVDs^4^. So et al showed that potato consumption is negatively associated with cardio-metabolic risk factors and disease.^5^ Since foods are usually consumed together and because it is likely interactions and synergif occurs between different nutrients, it is important to evaluate associations between different dietary patterns and CVDs.

To tease out the relative role of different plant-based foods (for example, fruits versus fruit juices), Satija et al created three types of plant-based dietary indices and examined these indices in relation to diabetes and CVD risk in the US.^6,7^ The advantage of Satija’s method ^6^ of categorizing plant-based dietary patterns over other methods (such as classification as vegetarian/non-vegitarian or according to the Dietary Approaches to Stop Hypertension (DASH) diet) is its emphasis on the quality of plant-based foods and their classification as healthy and less healthy plant foods. Few studies using this method have examined the association between plant-based dietary indices and health outcomes. In a general population of middle-aged adults, Kim et al found that higher adherence to a plant-based diet (measured with plant-based dietary indices) was associated with lower incidence of CVD, CVD mortality, and all-cause mortality^8^. However, they did not find any association between hPDI and CVD risk. They also failed to find a significant association between uPDI and these outcomes^8^. Another study by Kim et al in the US showed an inverse association between hPDI and all-cause mortality^9^. Heianza et al showed that hPDI may reduce the risk of CVDs, regardless of genetic susceptibility^10^. Other recent research by Waterplas et al in an adult Flemish population found few significant associations between changes in plant-based dietary indices (over ten years) and changes in anthropometric parameters and blood lipids.^11^ Because of differences between dietary components across countries, it is important to assess plant-based diets in different contexts. In Iran, a few studies have examined how plant-based dietary indices are associated with outcomes such as obesity, psychological disorders, sleep problems, gestational diabetes, and inflammatory biomarkers.^12-15^

To the best of our knowledge, no study has examined associations between plant-based diets and CVD risk factors using PDI, hPDI, and uPDI. Therefore, we sought to determine how these plant-based dietary indices were associated with CVD risk factors, such as high waist circumference, elevated Body Mass Index (BMI), dyslipidemia, and presense of a hypertriglyceridemic waist.

## Materials and Methods

This cross-sectional study was conducted with 371 women between 18-50 years old who attended ten health centers affiliated with Tehran University of Medical Sciences (TUMS) from Agust 2016 to March 2017. Our sample size was calculated using the following formula: $n=\frac{\left[{\left(z_{1}-\frac{\alpha }{2}\right)}^{2}s^{2}\right]}{d^{2}}$ ; (n = desired number of samples; Z_1_ = mean of variable; α = type one error; Z_1-α/2_ = standardized value for the corresponding level of confidence (95% CI), it is 1.96; d = margin of error or rate of precision; and s = SD which was based on a previous study or pilot study). We estimated the necessary sample size using information on high-density lipoprotein cholesterol (HDL-C) from Haghighatdoost et al’s article (HDL-C:46.7 ± 11; α: 0.05; d: 0.028 × 46.7).^16^

Using convenience sampling, participants were selected based on the population ratio of people accessing health center services. Informed written consent was given by all participants. Inclusion criteria were: age 18–50 years, Iranian (not immigrants), pre-menopausal, not pregnant or lactating, no current illness (e.g. diabetes, CVD, cancer, liver dysfunction, or kidney dysfunction)^17^. The study was approved by the Ethical Committee at TUMS (IRTUMS.VCR.REC.1397.431).

Usual dietary intake was assessed using a valid and reliable semi-quantitative 168-item FFQ^18^. The frequency of consumption of each food during the previous year was based on a daily, weekly, or monthly intake. Portion sizes of consumed foods were converted to grams using a household scale guide. Plant-based dietary indices were calculated using Satija et al’s method.^6^ After adjustment for energy according to nutrient resemblance, 19 food groups were created. These food groups were classified into three larger categories of healthy plant foods, less healthy plant foods, and animal foods. Plant foods were divided into healthy and less healthy, based on recent research investigating the association of foods and cardiovascular risk factors^6^. According to literature, foods that have adverse effects on cardiovascular health were categorized as less healthy foods and foods that have beneficial effects on cardiovascular health were classified as healthy foods. For example, a systematic review revealed that there was a significant association between white rice consumption and several cardiovascular disease risk factors, including type 2 diabetes and metabolic syndrome^19^. Another meta-analysis showed a dose-response relationship indicating that higher consumption of sugar-sweetened beverages was associated with a higher risk of hypertension and coronary heart diseases.^20^ In another population-based study, fruit and vegetable intakes were associated with reduced risk of CVD^4^.

Table 1 details some important foods making up each of the food groups. All food groups were categorized into deciles, and received positive or reverse scores from 1 to 10. To compute PDI, positive scores for all plant food groups and reverse scores for animal food groups were summed. To compute hPDI, healthy plant food groups were given positive scores, and less healthy plant food groups and animal food groups were given inverse scores. Finally, uPDI was calculated by assigning positive scores for less healthy plant food groups, and inverse scores for healthy plant food groups and animal food groups. Food group scores were summed to create the indices. Then, the indices were divided into tertiles.

**Table 1:** Examples of foods constituting the food groups

Plant Food Groups	Healthy	Whole Grains	Whole Grain, Cooked Oatmeal, Dark Bread, Oats
		Fruits	Apples, Raisins or Grapes, Prunes, Bananas, Pears Cantaloupe, Watermelon, Oranges, Peaches, Grapefruit, Strawberries, Apricots, Plums, Blueberries
		Vegetables	Broccoli, Cabbage, Cauliflower, Mixed Vegetables, Yellow or Winter Squash, Garlic, Eggplant or Zucchini, Spinach (Cooked or Raw), Kale or Mustard Orchard Greens, Head Lettuce, Romaine or Leaf Lettuce, Celery, Mushrooms, Corn, Tomatoes, Tomato Juice, Tomato Sauce, Carrots
		Nuts	Nuts, Peanut Butter
		Legumes	Bean or Lentils, String Beans, Soybeans, Peas, Mung
		Vegetable Oils	Vegetable Oil
		Whole Grains	Whole Grain, Dark Bread, Oat
	Less Healthy	Fruit Juices	Fruit Juice
		Refined Grains	Rice, White Bread (Lavash, Barbary, Taftoon), Muffins or Biscuits, Pancakes, Crackers, Pasta, Wheat, Flour
		Potatoes	Potato Chips, French Fries, Baked or Mashed Potatoes
		Sugar-Sweetened Beverages	Carbonated Beverages With Sugar, Non-Carbonated Fruit Drinks With Sugar
		Sweets And Desserts	Honey, Chocolates, Candy Bars, Jellies, Cookies (home-baked or ready-made), Brownies, Cake, Jams, Preserves or Syrup
		Hydrogenated Vegetable Oils	Margarine, Solid Oil
Animal Food Groups		Animal Fats	Butter
		Dairies	Ice Cream, Whole Milk, Cream, Skim Low-Fat Milk, Sherbet, Cheese
		Eggs	Eggs
		Seafood	Shrimp, Fish
		Meats	Chicken or Turkey, Processed Meats, Liver, Hamburger, Hot Dogs, Beef or Lamb
		Animal-Based Foods	Mayonnaise, Pizza, Chowder

Participant height was measured to the nearest millimeter using a stiff measuring rod while participants stood in a normal position with their shoes off. Weight was calculated to the nearest 0.1 kg using a digital scale (SECA, Hamburg, Germany), while participants were wearing minimal clothing and no shoes. BMI was calculated as weight in kilograms divided by height in meters squared. BMI was dichotomized with BMI ≥ 25 indicating overweight/obese compared to the rest of the sample. Waist circumference (WC) was measured to the nearest millimeter at the midpoint of the last rib and the iliac crest using an inflexible tape. A WC of > 88 cm was considered abdominal obesity.

Twelve-hour fasting blood samples (10 mL) were collected. Serum levels of FBS, total cholesterol (TC), low-density lipoprotein cholesterol (LDL-C), high-density lipoprotein cholesterol (HDL-C), and triglycerides (TG) were measured using enzymatic reagents (Pars Azmoon, Tehran, Iran) adapted to an auto-analyzer system (Selectra E, Vitalab, Holliston, the Netherlands). Systolic and diastolic blood pressures (SBP and DBP) were measured twice using a standard mercury sphygmomanometer (ALPK2, JAPAN) after participants rested in a seated position for 15 minutes. The average of two measurements was reported. According to the Adult Treatment Panel III (ATP III), FBS ≥ 110 mg/dL; WC > 88 cm; SBP ≥ 130 mmHg or DBP ≥ 85mm Hg; Serum TG ≥ 150 mg/dL; TC ≥ 200 mg/dL; serum LDL-C ≥ 130 mg/dL; and serum HDL-C < 50 mg/dL were considered CVD risk factors^21^.

Demographic data, smoking habits, medical history, and information on medications were collected through face-to-face interviews by skilled research staff. Socioeconomic status was determined through several questions about the number of family members in the household, occupation, education, income, homeownership, the number of rooms in the home, having modern furniture in the household, car ownership, and travel outside and within the country. All of these variables were obtained through a general demographic questionnaire administered by a trained interviewer.

Participants were also asked to record their daily activities for a 24-hour period. An individual’s mean physical activity level was calculated using the following equation: PA mean = ∑ (MET*Time for each activity). PA mean corresponded to the mean amount of time one engaged in physical activity. Time for each activity refers to the total time spent on each activity in a day. MET is the metabolic equivalent task as defined by Ainsworth et al. ^22^

To identify participants with hypertriglyceridemic waists (HTGW), participants were categorized into four phenotypes based on a study by Esmaillzadeh et al^23^: *1*) high serum TG and high WC (TG ≥ 150 mg/dL and WC ≥ 79 cm); *2*) low serum TG and high WC (TG < 150 mg/dL and WC ≥ 79 cm); *3*) high serum TG and low WC (TG ≥ 150 mg/dL and WC < 79 cm); and *4*) low serum TG and low WC (TG < 150 mg/dL and WC < 79 cm). All of our study participants fell into the first or second categories.

### Statistical analysis

The Kolmogorov-Smirnov test was conducted to check for normality of the variables. Chi-square and analysis of variance (one-way ANOVA) tests were used to compare participants’ qualitative and quantitative characteristics across the different tertiles of adherence to the PDI. Analysis of covariance was used to evaluate the association between adherence to plant-based dietary indices and dietary intakes as well as biochemical tests. All dietary intakes including macro- and micro-nutrients as well as foods and food groups were adjusted for energy intake. The mean and standard error of cardiovascular risk factors for tertiles of plant-based dietary indices were determined by the ANCOVA test in a crude model (Model 1) and in an adjusted model (Model 2).

In the second model, age, energy intake, SES, physical activity, use of estrogen, medication, supplement consumption, and BMI were included as confounders. Logistic regression was performed to evaluate the associations between the scores for adherence to PDIs and CVD risk factors in both crude and adjusted models. All statistical analyses were performed using SPSS, version 16 (SPSS Inc., Chicago, IL, USA), and *P* < 0.05 was used as a cutoff to indicate statistical significance.

## Results

Participants’ general characteristics are displayed in Table 2. Women with the highest PDI scores were more active than those with lower scores (*P* < 0.0001) and were more likely to take supplements less than once per month (*P* = 0.002). Participants in the top tertile of hPDI were older (*P* < 0.0001), more active (*P* < 0.0001), and took fewer medications (*P* = 0.003) and supplements (*P* = 0.002) compared to participants in the lowest tertile. Furthermore, a higher uPDI score was associated with lower engagement in physical activity (*P* = 0.034), taking more medications (*P* = 0.035), and being younger compared to those in the lowest tertile (*P* = 0.003). No additional differences were seen in participant baseline variables across tertiles of PDI, hPDI, and uPDI.

**Table 2:** General characteristics of study participants across tertiles of the plant-based diet index (PDI), the healthy plant-based diet index (hPDI), and the unhealthy plant-based diet index (uPDI)

**Variables**	**PDI**			***P*** **value** ^b^	**hPDI**			***P*** ** value**	**uPDI**			***P*** **-value**
	**Tertile 1 (121)**	**Tertile 2 (124)**	**Tertile 3 (126)**		**Tertile 1 (125)**	**Tertile 2 (120)**	**Tertile 3 (126)**		**Tertile 1 (125)**	**Tertile 2 (118)**	**Tertile 3 (128)**	
Age^a^ (year)	30.75 ± 6.63	29.79 ± 7.01	31.46 ± 7.08	0.161	28.32 ± 5.12	30.12 ± 6.67	33.52 ± 7.72	< 0.0001	32.38 ± 7.59	29.80 ± 6.67	29.80 ± 6.18	0.003
Physical Activity (Met.h.d^-1^)	26.1 ± 2.92	28.50 ± 5.01	28.06 ± 3.86	< 0.0001	24.85 ± 4.04	27.02 ± 3.51	28.80 ± 4.56	< 0.0001	27.86 ± 4.16	28.09 ± 3.99	26.81 ± 4.21	0.034
Waist^a^ (cm)	88.51 ± 6.76	89.71 ± 7.99	88.49 ± 6.28	0.298	88.16 ± 6.10	89.22 ± 7.18	89.35 ± 7.75	0.345	88.60 ± 7.01	88.82 ± 7.14	89.28 ± 7.04	0.736
BMI^a^(kg/m)	23.79 ± 4.28	24.69 ± 3.87	24.29 ± 3.95	0.223	23.82 ± 4.25	24.47 ± 3.99	24.50 ± 3.87	0.329	24.21 ± 3.68	24.21 ± 3.93	24.36 ± 4.48	0.944
SES				0.12				0.066				0.104
Poor	29(24)	37(29.8)	40(31.7)		38(30.4)	30(25)	38(30.2)		29(23.2)	44(37.3)	33(25.8)	
Moderate	44(36.4)	41(33.1)	54(42.9)		35(28.0)	53(44.2)	51(40.5)		53(42.4)	40(33.9)	46(35.9)	
Rich	48(39.7)	46(37.1)	32(25.4)		52(41.6)	37(30.8)	37(29.4)		43(34.4)	34(28.8)	49(38.3)	
Supplement Consumption n (%)				0.002				0.002				0.192
No	39(32.2)	61(49.2)	67(53.2)		41(32.8)	65(54.2)	61(48.4)		64(51.2)	47(39.8)	56(43.8)	
Yes	82(67.8)	63(50.8)	59(46.8)		84(67.2)	55(45.8)	65(51.6)		61(48.8)	71(60.2)	72(56.2)	
Medication n(%)				0.633				0.003				0.035
No	99(81.8)	107(86.3)	106(84.1)		95(76)	110(91.7)	107(84.9)		109(87.2)	104(88.1)	99(77.3)	
Yes	22(18.2)	17(13.7)	20(15.9)		30(24.0)	10(8.3)	19(15.1)		16(12.8)	14(11.9)	29(22.7)	
HTGW n (%)				0.473				0.448				0.752
^c^Cut1	19(15.7)	14(11.3)	14(11.1)		14(11.2)	19(15.8)	14(15.1)		14(11.2)	17(14.4)	16(12.5)	
Cut2	102(84.3)	110(88.7)	112(88.9)		111(88.8)	101(84.2)	112(88.9)		111(88.8)	101(85.6)	112(87.5)	

Abbreviations: SES, socioeconomic status; PDI, overall plant-based diet index; hPDI, healthful plant-based diet index; uPDI, unhealthful plant-based diet index; HTGW, hypertriglyceridemic waist; BMI, body mass index; SES,socio economic status ^a^Values are mean (SD) for continuous variables and number (percentage) for dichotomous variables, ^b^P-values were calculated using one-way ANOVA for continuous variables and chi-square tests for categorical variables.^c^HTGW: Cut1 (TG < 150; WC > 79); Cut2 (TG > 150; WC > 79)

**Figure 1:**
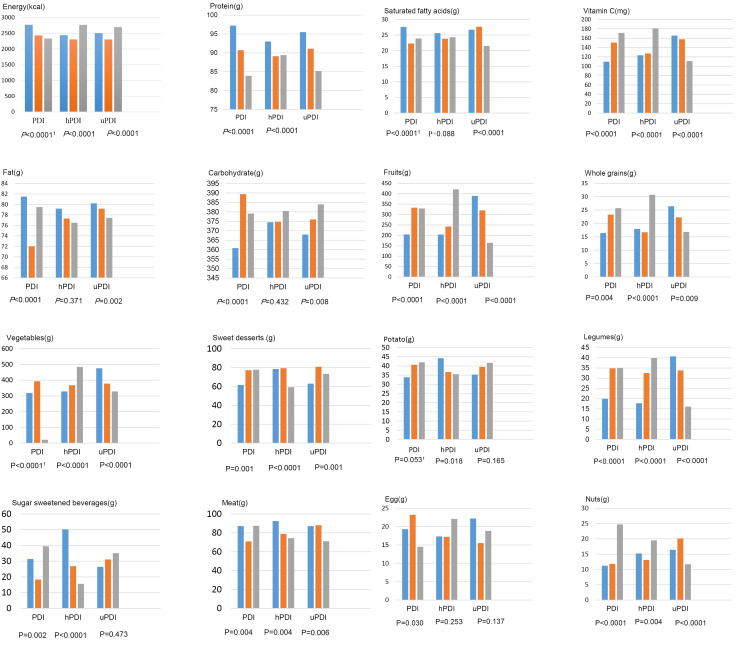
Participants’ dietary intakes across categories of PDI, hPDI, and uPDI

Participants’ dietary intakes across categories of PDI, hPDI, and uPDI are shown in Figure 1. Participants with the highest PDI scores consumed less energy, protein, fat, saturated fatty acids, dairy, egg, meats, SFAs, compared with those in the lowest tertile (*P* < 0.05). However, they consumed more carbohydrates, vitamin C, fruits, legumes, nuts and sweets, and desserts (*P* < 0.05).

Furthermore, higher intakes of total energy, vitamin C, fruits, egg, legumes, and nuts were observed in participants with diets categorized in the highest tertile of hPDI relative to the lowest tertile (*P* < 0.05). Lower intakes of SFAs, sweets and desserts, and meats were found among participants with diets in the highest tertile compared to in the lowest tertile of hPDI (*P* < 0.05). Finally, higher uPDI scores were associated with higher intakes of total energy, carbohydrate, and sweets/desserts (*P* < 0.0001) and with lower intakes of protein, fat, SFAs, vitamin C, fruits, legumes, nuts, dairy, and meat (*P* < 0.05).

Cardiovascular risk factors within tertiles of plant-based dietary indices are shown in Table 3. Among the associations we investigated, only a few were statistically significant. After adjustment for age, SES, physical activity, taking medications, supplement consumption, energy intake, and BMI, the associations were attenuated and only PDI and LDL-C remained statistically significant (79.61 ± 14.36 mg/dL vs. 83.01 ± 14.96 mg/dL; *P* = 0.021). Furthermore, after adjustment higher uPDI was associated with higher TG (101.5 ± 56.55 mg/dL vs. 97.70 ± 56.46 mg/dL; *P* < 0.0001).

**Table 3:** Cardiovascular risk factors by tertile of plant-based dietary indices

**Variables**	**PDI**			***P*** ** value**	**hPDI**			***P*** ** value**	**uPDI**			***P*** ** value**
	**Tertile 1 (n=121)**	**Tertile 2 (n=124)**	**Tertile 3 (n=126)**		**Tertile 1 (n=125)**	**Tertile 2 (n=120)**	**Tertile 3 (n=126)**		**Tertile 1 (n=125)**	**Tertile 2 (n=118)**	**Tertile 3 (n=128)**	
WC(cm)												
Model1^a^	88.51 ± 7.04	89.71 ± 7.01	88.49 ± 7.07	0.298	88.16 ± 7.04	89.22 ± 7.01	89.35 ± 7.07	0.345	88.60 ± 7.04	88.82 ± 7.06	89.28 ± 7.01	0.736
Model2^b^	88.47 ± 7.48	89.74 ± 7.13	88.50 ± 7.18	0.289	88.30 ± 7.50	88.99 ± 7.23	89.42 ± 7.67	0.519	88.49 ± 7.16	88.89 ± 7.28	89.33 ± 7.24	0.656
BMI(kg/m^2^)												
Model1	23.79 ± 4.07	24.69 ± 4.01	24.29 ± 4.03	0.223	23.82 ± 4.02	24.47 ± 4.05	24.50 ± 4.04	0.329	24.21 ± 4.04	24.21 ± 4.02	24.36 ± 4.07	0.944
Model2	23.82 ± 4.29	24.69 ± 4.12	24.27 ± 4.15	0.291	23.76 ± 4.25	24.38 ± 4.16	24.64 ± 4.38	0.284	24.22 ± 4.14	24.11 ± 4.13	24.43 ± 4.18	0.832
FBS (mg/dL)												
Model 1	89.63 ± 9.35	87.09 ± 9.36	89.70 ± 9.31	0.044	87.22 ± 9.17	87.43 ± 9.20	91.68 ± 9.20	< 0.0001	90.30 ± 9.39	87.64 ± 9.33	88.41 ± 9.39	0.075
Model 2	89.43 ± 8.91	87.48 ± 8.57	89.50 ± 8.53	0.11	88.58 ± 8.94	88.33 ± 8.65	89.48 ± 9.09	0.591	89.47 ± 8.61	88.52 ± 8.69	88.42 ± 8.60	0.575
SBP (mmHg)												
Model 1	114.09 ± 9.90	114.09 ± 9.91	113.93 ± 9.87	0.634	113.92 ± 9.95	114.21 ± 9.96	114.92 ± 9.87	0.713	114.68 ± 9.84	113.22 ± 9.88	115.08 ± 9.84	0.306
Model 2	114.3 ± 10.34	114.8 ± 9.91	114.0 ± 9.87	0.824	114.3 ± 10.29	113.9 ± 9.96	114.8 ± 10.43	0.787	114.6 ± 9.95	113.5 ± 9.99	115.0 ± 9.95	0.474
DBP (mmHg)												
Model 1	78.64 ± 6.05	78.31 ± 6.02	77.86 ± 6.05	0.592	78.00 ± 6.04	78.13 ± 8.21	78.65 ± 8.30	0.661	78.28 ± 6.04	78.05 ± 5.97	78.44 ± 5.99	0.88
Model 2	78.74 ± 6.27	78.11 ± 8.24	77.95 ± 6.40	0.596	78.26 ± 6.26	78.07 ± 6.02	78.45 ± 6.28	0.891	78.25 ± 6.04	78.33 ± 5.97	78.21 ± 5.99	0.989
TG (mg/dL)												
Model 1	102.25 ± 57.97	98.00 ± 57.9	99.98 ± 57.90	0.848	96.54 ± 57.91	102.14 ± 57.93	101.57 ± 57.90	0.704	98.52 ± 58.02	99.93 ± 57.99	101.68 ± 57.91	0.91
Model 2	102.1 ± 58.63	97.02 ± 56.48	101.1 ± 56.44	0.763	99.22 ± 58.58	101.4 ± 56.72	99.61 ± 59.69	0.95	97.70 ± 56.46	101.0 ± 56.80	101.5 ± 56.55	< 0.0001
TC (mg/dL)												
Model 1	174.54 ± 34.54	174.91 ± 34.53	172.94 ± 34.56	0.891	172.82 ± 34.54	172.89 ± 34.49	176.57 ± 34.56	0.618	171.34 ± 34.43	172.42 ± 34.43	178.40 ± 34.38	0.216
Model 2	172.6 ± 36.19	175.4 ± 34.87	174.4 ± 34.89	0.834	172.8 ± 36.11	173.8 ± 34.93	175.7 ± 36.80	0.836	171.8 ± 34.77	174.5 ± 34.97	177.0 ± 34.83	0.487
LDL-C (mg/dL)												
Model 1	83.80 ± 14.96	76.97 ± 14.93	79.54 ± 14.81	0.002	79.31 ± 14.98	77.89 ± 15.00	82.90 ± 15.03	0.027	80.63 ± 14.98	76.62 ± 14.99	82.70 ± 14.93	0.006
Model 2	83.01 ± 14.96	77.67 ± 14.37	79.61 ± 14.36	0.021	80.60 ± 14.98	78.20 ± 14.56	81.33 ± 15.26	0.229	79.52 ± 14.42	78.59 ± 14.55	81.98 ± 14.48	0.172
HDL-C (mg/dL)												
Model 1	46.91 ± 9.46	48.25 ± 9.47	48.66 ± 9.42	0.314	48.87 ± 9.39	46.89 ± 9.42	48.05 ± 9.42	0.256	49.06 ± 9.39	46.90 ± 9.45	47.84 ± 9.39	0.201
Model 2	47.20 ± 9.68	48.06 ± 9.36	48.57 ± 9.31	0.546	48.52 ± 9.73	46.88 ± 9.42	48.41 ± 9.87	0.319	49.21 ± 9.28	46.38 ± 9.34	48.18 ± 9.27­­­	0.063

Abbreviations: PDI, plant-based diet index; hPDI, healthful plant-based diet index; uPDI, unhealthful plant-based diet index; WC,waist circumference; BMI,body mass index; FBS, fasting blood sugar; SBP, systolic blood pressure; DBP, diastolic blood pressure; TG, triglyceride; TC, total cholesterol; LDL-C, low density cholesterol; HDL-C, high density cholesterol. Values are presented as mean ± SD;P-values were calculated using ANCOVA. ^a^Model 1: Crude model ^b^Model 2: Adjusted for age, BMI,energy intake, socioeconomic status, and physical activity, taking medication, and supplement consumption

Multivariate-adjusted odds ratios (OR) and 95% conﬁdence intervals (CIs) for CVD risk factors of PDI, hPDI, and uPDI are presented by tertile in Table 4. There was no significant association between plant-based diet indices and CVD risk factors. Only higher PDI was associated with increased risk of low HDL-C in a crude model (OR: 0.60, 95% CI: 0.35-0.99, *P* = 0.045). This association disappeared after adjustment for age, BMI, energy intake, socioeconomic status, physical activity, medication, and supplement consumption (OR: 0.61, 95% CI: 0.35-1.05, *P* = 0.062).

**Table 4:** Odds ratios (OR) and 95% conﬁdence intervals (CIs) for cardiovascular risk factors among tertiles of plant-based dietary indices

**Variables**	**PDI**			***P*** **value** ^a^	**hPDI**			***P*** ** value**	**uPDI**			***P*** ** value**
	**Tertile 1 (n=121)**	**Tertile 2 (n=124)**	**Tertile 3 (n=126)**		**Tertile 1 (125)**	**Tertile 2 (120)**	**Tertile 3 (126)**		**Tertile 1 (n=125)**	**Tertile 2 (n=118)**	**Tertile 3 (n=128)**	
WC(cm)												
Model 1^a^	1	1.43(0.82-2.50)	1.21(0.69-2.11)	0.527	1	1.29(0.74-2.25	1.20(0.69-2.09)	0.514	1	1.28(0.73-2.25)	1.53(0.89-2.65)	0.126
Model 2^b^	1	1.60(0.88-2.89)	1.31(0.72-2.39)	0.571	1	1.14(0.64-2.03)	1.12(0.61-2.09)	0.707	1	1.40(0.78-2.53)	1.63(0.92-2.87)	0.093
TG (mg/dL)												
Model 1	1	0.68(0.33-1.43)	0.67(0.32-1.41)	0.282	1	1.49(0.71-3.13)	0.99(0.45-2.18)	0.981	1	1.34(0.63-2.85)	1.13(0.53-2.43)	0.76
Model 2	1	0.57(0.24-1.35)	0.68(0.29-1.61)	0.406	1	1.54(0.67-3.53)	0.92(0.37-2.32)	0.896	1	1.39(0.61-3.16)	1.07(0.46-2.46)	0.881
TC (mg/dL)												
Model 1	1	0.88(0.47-1.63)	0.90(0.49-1.68)	0.75	1	1.05(0.56-1.98)	1.15(0.62-2.13)	0.66	1	1.14(0.59-2.19)	1.65(0.89-3.06)	0.105
Model 2	1	1.15(0.59-2.25)	1.22(0.62-2.38)	0.575	1	1.11(0.57-2.18)	1.11(0.55-2.24)	0.772	1	1.18(0.59-2.38)	1.59(0.83-3.04)	0.157
HDL-C (mg/dL)												
Model 1	1	0.88(0.53-1.47)	0.60(0.36-0.99)	0.045	1	1.49(0.90-2.48)	0.89(0.55-1.47)	0.652	1	1.70(1.02-2.84)	1.27(0.77-2.08)	0.809
Model 2	1	0.94(0.54-1.64)	0.61(0.35-1.05)	0.062	1	1.46(0.85-2.51)	0.78(0.44-1.37)	0.417	1	1.94(1.13-3.33	1.22(0.72-2.05)	0.454
BMI												
Model 1	1	1.32(0.78-2.22)	1.24(0.74-2.09)	0.422	1	1.23(0.7-2.07)	1.39(0.83-2.32)	0.211	1	0.95(0.57-1.60)	1.06(0.64-1.77)	0.809
Model 2	1	1.29(0.74-2.24)	1.19(0.68-2.08)	0.44	1	1.22(0.71-2.11)	1.59(0.89-2.83)	0.119	1	0.91(0.53-1.57)	1.06(0.63-1.79)	0.832
HTGW(TG ≥ 150; WC ≥ 79)												
Model 1	1	0.68(0.33-1.43)	0.67(0.32-1.41)	0.282	1	1.49(0.71-3.13)	0.99(0.45-2.18)	0.981	1	1.34(0.63-2.85)	1.13(0.53-2.43)	0.76
Model 2	1	0.72(0.32-1.61)	0.75(0.34-1.70)	0.504	1	1.60(0.73-3.47)	1.07(0.45-2.57)	0.838	1	1.34(0.61-1.93)	1.19(0.54-2.63)	0.675

PDI, plant-based diet index; hPDI, healthful plant-based diet index; uPDI, unhealthful plant-based diet index;WC,waist circumference; BMI,body mass index; FBS, fasting blood sugar; SBP: systolic blood pressure; DBP,diastolic blood pressure; TG, triglyceride; TC, total cholesterol; LDL-C,low density cholesterol; HDL-C, high density cholesterol. *P* values were calculated using logistic regression models.; ^a^Model 1: crude model ^b^Model 2: adjusted for age, BMI, energy intake, socioeconomic status, and physical activity, taking medication, and supplements consumption.

## Discussion

**This study aimed to investigate that how plant dietary indices (overall PDI, hPDI, and uPDI) are associated with anthropometric parameters and blood lipids in Iranian women. We failed to find significant associations between plant-based diet indices and anthropometric parameters such as BMI and WC even after adjustment.** In contrast to our results, Waterplas et al reported that higher PDI scores were associated with an increase in BMI in adjusted analyses.^11^ Chen et al found that higher PDI was associated with lower BMI, waist circumference, body fat percentage, and fat mass index^24^. In a cross-sectional study by Zamani et al, higher uPDI was associated with a higher risk of obesity, but PDI and hPDI were not^12^.

Bolori et al found that higher hPDI could be useful to reduce inflammatory biomarkers like TGF-b and hs-CRP^15^. However, uPDI did not significantly increase these inflammatory markers^15^. In a crude model, we found that higher PDI was associated with decreased risk of low HDL-C level in Iranian women but that this association disappeared after adjustment for potential confounders.

In addition, some associations between plant-based dietary indices and lipid levels were observed. Higher PDI was associated with lower LDL-C in unadjusted analyses. In contrast, in adjusted models, higher hPDI and uPDI were associated with a higher concentration of LDL-C. In our crude model, a positive association was found between PDI / hPDI and FBS. All of these associations disappeared after adjustment for potential confounders. Only the association between PDI and LDL-C remained significant after adjustment. Moreover, a positive association was observed between TG concentration and uPDI in the adjusted model.

In a crude model, Waterplas et al found a significant positive association between uPDI and TC level^11^. However, they found no significant associations between plant-based dietary indices and TG, HDL-C, and LDL-C ^11^. However, Yokoyama et al reported beneficial effects of plant-based diets on TC, LDL-C, HDL-C ^25^.

The beneficial effects of several dietary patterns that emphasize plant foods (such as the Mediterranean diet and DASH diet) on CVD risk factors have been shown in previous studies.^26-28^ An advantage of the methods we used compared to those of most prior studies was the categorization of foods into animal foods, healthy plant foods, and less healthy plant foods, based on their main nutrients. In other words, our approach considered the nature of plant food groups.

Some nutrients such as n-3 polyunsaturated fatty acids and some proteins (essential and easily digestible amino acids) are likely to be related to significant increases in HDL cholesterol^29^. High fructose in sweets and desserts may lead to hypertriglyceridemia and insulin resistance.^30^

The soluble fiber in oat and beans may cause a modest decrease in LDL-C by altering cholesterol synthesis, lowering cholesterol absorption as well as by increasing bile acid synthesis and decreasing bile acid absorption^31^. Polyphenols in several plant foods such as fruits, vegetables, tea, coffee, nuts, and vegetable oils might play a role in limiting LDL oxidation and improve the lipid profile^32^.

In addition, findings are conflicting regarding the association between rice (a less healthy food) and CVD risk factors. Eating patterns probably have an impact on the association between foods like rice and CVD risk factors.^33-35^ Shan et al compared adherence to the Healthy Eating Index–2015 (HEI-2015), the Alternate Mediterranean Diet Score (AMED), the Healthful Plant-Based Diet Index (HPDI), and the Alternate Healthy Eating Index (AHEI) on the risk of CVD over 32 years of follow-up. They found that all of these dietary indices were consistently associated with a lower risk of CVD^36^.

To the best of our knowledge, this is the first study to investigate the associations between plant-based dietary indices and cardiovascular risk factors in Iranian women. Strengths of the current study include a sufficient sample size to provide adequate power, similarities in participant ages, and a focus on women.

A main limitation of this study was its cross-sectional design. Future studies using cohort and case-control designs should be carried out to confirm these findings. Moreover, the sampling method may explain the fact we discovered no associations between plant-based dietary indices and cardiovascular risk factors. Another limitation was the use of FFQs to assess dietary intake. Since FFQ retrospectively assesses dietary intake, recall of dietary intake might have been imperfect and consequently have led to misclassification. Residual confounding is also inevitable. Some foods such as salt and salty foods weren’t considered in these indices. In addition, all animal-based foods in these indices were reverse scored. However, some animal foods, such as fish and dairy products, also show positive favorable effects on cardiovascular health.^37-39^

## Conclusion

We observed no significant association between plant-based dietary indices and risk of low HDL-C. In adjusted models, only higher PDI was associated with a decreased concentration of LDL-C and higher uPDI was associated with an increase in TG level. No other associations were found between plant-based dietary patterns and cardiovascular risk factors. Future studies with longitudinal designs are needed to conﬁrm these ﬁndings.

## Acknowledgments

This study was based on a Masters of Science dissertation approved by the School of Nutritional Sciences and Dietetics, Tehran University of Medical Sciences, Tehran, Iran (Thesis code: 9611323005).

## Funding

This study was supported by Tehran University of Medical Sciences (grant number: 9611323005).

## Ethical approval

This study was approved by the ethical Committee at TUMS (IRTUMS.VCR.REC.1397.431). Written informed consent and was obtained from participants.

## Competing interest

The authors declare that they have no conflicts of interest.

## References

[1] World Health Organization (WHO). The Top 10 Causes of Death. WHO; 2018.

[2] World Health Organization (WHO). The Top 10 Causes of Death. WHO; 2014.

[3] Zurbau A, Au-Yeung F, Blanco Mejia S, Khan TA, Vuksan V, Jovanovski E (2020). Relation of different fruit and vegetable sources with incident cardiovascular outcomes: a systematic review and meta-analysis of prospective cohort studies. J Am Heart Assoc.

[4] Aune D, Giovannucci E, Boffetta P, Fadnes LT, Keum N, Norat T (2017). Fruit and vegetable intake and the risk of cardiovascular disease, total cancer and all-cause mortality-a systematic review and dose-response meta-analysis of prospective studies. Int J Epidemiol.

[5] So J, Avendano EE, Raman G, Johnson EJ (2020). Potato consumption and risk of cardio-metabolic diseases: evidence mapping of observational studies. Syst Rev.

[6] Satija A, Bhupathiraju SN, Rimm EB, Spiegelman D, Chiuve SE, Borgi L (2016). Plant-based dietary patterns and incidence of type 2 diabetes in US men and women: results from three prospective cohort studies. PLoS Med.

[7] Satija A, Bhupathiraju SN, Spiegelman D, Chiuve SE, Manson JE, Willett W (2017). Healthful and unhealthful plant-based diets and the risk of coronary heart disease in US adults. J Am Coll Cardiol.

[8] Kim H, Caulfield LE, Garcia-Larsen V, Steffen LM, Coresh J, Rebholz CM (2019). Plant‐based diets are associated with a lower risk of incident cardiovascular disease, cardiovascular disease mortality, and all‐cause mortality in a general population of middle‐aged adults. J Am Heart Assoc.

[9] Kim H, Caulfield LE, Rebholz CM (2018). Healthy plant-based diets are associated with lower risk of all-cause mortality in US adults. J Nutr.

[10] Heianza Y, Zhou T, Sun D, Hu FB, Manson JE, Qi L (2020). Genetic susceptibility, plant-based dietary patterns, and risk of cardiovascular disease. Am J Clin Nutr.

[11] Waterplas J, Versele V, D’Hondt E, Lefevre J, Mertens E, Charlier R (2020). A 10-year longitudinal study on the associations between changes in plant-based diet indices, anthropometric parameters and blood lipids in a Flemish adult population. Nutr Diet.

[12] Zamani B, Daneshzad E, Siassi F, Guilani B, Bellissimo N, Azadbakht L (2020). Association of plant-based dietary patterns with psychological profile and obesity in Iranian women. Clin Nutr.

[13] Zamani B, Milajerdi A, Tehrani H, Bellissimo N, Brett NR, Azadbakht L (2019). Association of a plant-based dietary pattern in relation to gestational diabetes mellitus. Nutr Diet.

[14] Daneshzad E, Keshavarz SA, Qorbani M, Larijani B, Bellissimo N, Azadbakht L (2020). Association of dietary acid load and plant-based diet index with sleep, stress, anxiety and depression in diabetic women. Br J Nutr.

[15] Bolori P, Setaysh L, Rasaei N, Jarrahi F, Yekaninejad MS, Mirzaei K (2019). Adherence to a healthy plant diet may reduce inflammatory factors in obese and overweight women-a cross-sectional study. Diabetes Metab Syndr.

[16] Haghighatdoost F, Sarrafzadegan N, Mohammadifard N, Sajjadi F, Maghroon M, Boshtam M (2013). Healthy eating index and cardiovascular risk factors among Iranians. J Am Coll Nutr.

[17] Saraf-Bank S, Haghighatdoost F, Esmaillzadeh A, Larijani B, Azadbakht L (2017). Adherence to Healthy Eating Index-2010 is inversely associated with metabolic syndrome and its features among Iranian adult women. Eur J Clin Nutr.

[18] Asghari G, Rezazadeh A, Hosseini-Esfahani F, Mehrabi Y, Mirmiran P, Azizi F (2012). Reliability, comparative validity and stability of dietary patterns derived from an FFQ in the Tehran Lipid and Glucose Study. Br J Nutr.

[19] Izadi V, Azadbakht L (2015). Is there any association between rice consumption and some of the cardiovascular diseases risk factors? a systematic review. ARYA Atheroscler.

[20] Xi B, Huang Y, Reilly KH, Li S, Zheng R, Barrio-Lopez MT (2015). Sugar-sweetened beverages and risk of hypertension and CVD: a dose-response meta-analysis. Br J Nutr.

[21] Evaluation Evaluation (2002). Third report of the National Cholesterol Education Program (NCEP) Expert Panel on detection, evaluation, and treatment of high blood cholesterol in adults (Adult Treatment Panel III) final report. Circulation.

[22] Ainsworth BE, Haskell WL, Whitt MC, Irwin ML, Swartz AM, Strath SJ (2000). Compendium of physical activities: an update of activity codes and MET intensities. Med Sci Sports Exerc.

[23] Esmaillzadeh A, Mirmiran P, Azizi F (2005). Whole-grain intake and the prevalence of hypertriglyceridemic waist phenotype in Tehranian adults. Am J Clin Nutr.

[24] Chen Z, Schoufour JD, Rivadeneira F, Lamballais S, Ikram MA, Franco OH (2019). Plant-based diet and adiposity over time in a middle-aged and elderly population: the Rotterdam Study. Epidemiology.

[25] Yokoyama Y, Levin SM, Barnard ND (2017). Association between plant-based diets and plasma lipids: a systematic review and meta-analysis. Nutr Rev.

[26] Siervo M, Lara J, Chowdhury S, Ashor A, Oggioni C, Mathers JC (2015). Effects of the Dietary Approach to Stop Hypertension (DASH) diet on cardiovascular risk factors: a systematic review and meta-analysis. Br J Nutr.

[27] Paula Bricarello L, Poltronieri F, Fernandes R, Retondario A, de Moraes Trindade EBS, de Vasconcelos FAG (2018). Effects of the Dietary Approach to Stop Hypertension (DASH) diet on blood pressure, overweight and obesity in adolescents: a systematic review. Clin Nutr ESPEN.

[28] Rosato V, Temple NJ, La Vecchia C, Castellan G, Tavani A, Guercio V (2019). Mediterranean diet and cardiovascular disease: a systematic review and meta-analysis of observational studies. Eur J Nutr.

[29] Wergedahl H, Liaset B, Gudbrandsen OA, Lied E, Espe M, Muna Z (2004). Fish protein hydrolysate reduces plasma total cholesterol, increases the proportion of HDL cholesterol, and lowers acyl-CoA: cholesterol acyltransferase activity in liver of Zucker rats. J Nutr.

[30] Faeh D, Minehira K, Schwarz JM, Periasamy R, Park S, Tappy L (2005). Effect of fructose overfeeding and fish oil administration on hepatic de novo lipogenesis and insulin sensitivity in healthy men. Diabetes.

[31] Smith CE, Tucker KL (2011). Health benefits of cereal fibre: a review of clinical trials. Nutr Res Rev.

[32] Quiñones M, Miguel M, Aleixandre A (2013). Beneficial effects of polyphenols on cardiovascular disease. Pharmacol Res.

[33] Khosravi-Boroujeni H, Sarrafzadegan N, Mohammadifard N, Sajjadi F, Maghroun M, Asgari S (2013). White rice consumption and CVD risk factors among Iranian population. J Health Popul Nutr.

[34] Kolahdouzan M, Khosravi-Boroujeni H, Nikkar B, Zakizadeh E, Abedi B, Ghazavi N (2013). The association between dietary intake of white rice and central obesity in obese adults. ARYA Atheroscler.

[35] Ahn Y, Park SJ, Kwack HK, Kim MK, Ko KP, Kim SS (2013). Rice-eating pattern and the risk of metabolic syndrome especially waist circumference in Korean Genome and Epidemiology Study (KoGES). BMC Public Health.

[36] Shan Z, Li Y, Baden MY, Bhupathiraju SN, Wang DD, Sun Q (2020). Association between healthy eating patterns and risk of cardiovascular disease. JAMA Intern Med.

[37] Vázquez C, Botella-Carretero JI, Corella D, Fiol M, Lage M, Lurbe E (2014). White fish reduces cardiovascular risk factors in patients with metabolic syndrome: the WISH-CARE study, a multicenter randomized clinical trial. Nutr Metab Cardiovasc Dis.

[38] Zhubi-Bakija F, Bajraktari G, Bytyçi I, Mikhailidis DP, Henein MY, Latkovskis G (2021). The impact of type of dietary protein, animal versus vegetable, in modifying cardiometabolic risk factors: a position paper from the International Lipid Expert Panel (ILEP). Clin Nutr.

[39] Dehghan M, Mente A, Rangarajan S, Sheridan P, Mohan V, Iqbal R (2018). Association of dairy intake with cardiovascular disease and mortality in 21 countries from five continents (PURE): a prospective cohort study. Lancet.

